# Outpatient Management of Children With World Health Organization Chest Indrawing Pneumonia: Implementation Risks and Proposed Solutions

**DOI:** 10.1093/cid/cix543

**Published:** 2017-06-09

**Authors:** Eric D McCollum, Amy Sarah Ginsburg

**Affiliations:** 1 Eudowood Division of Pediatric Respiratory Sciences, Johns Hopkins School of Medicine and Department of International Health, Johns Hopkins Bloomberg School of Public Health, Baltimore, Maryland; and; 2 Save the Children, Seattle, Washington

**Keywords:** World Health Organization Integrated Management of Child Illness guidelines, child pneumonia, chest indrawing, pulse oximetry, malnutrition

## Abstract

This Viewpoints article details our recommendation for the World Health Organization Integrated Management of Childhood Illness guidelines to consider additional referral or daily monitoring criteria for children with chest indrawing pneumonia in low-resource settings. We review chest indrawing physiology in children and relate this to the risk of adverse pneumonia outcomes. We believe there is sufficient evidence to support referring or daily monitoring of children with chest indrawing pneumonia and signs of severe respiratory distress, oxygen saturation <93% (when not at high altitude), moderate malnutrition, or an unknown human immunodeficiency virus (HIV) status in an HIV-endemic setting. Pulse oximetry screening should be routine and performed at the earliest point in the patient care pathway as possible. If outpatient clinics lack capacity to conduct pulse oximetry, nutritional assessment, or HIV testing, then we recommend considering referral to complete the evaluation. When referral is not possible, careful daily monitoring should be performed.

## Teaser

World Health Organization Integrated Management of Child Illness guidelines should consider the following revisions for children with chest indrawing pneumonia: referral or daily monitoring with signs of severe respiratory distress, oxygen saturation <93%, moderate malnutrition, or unknown HIV status (when HIV endemic).

The World Health Organization (WHO) Integrated Management of Childhood Illness (IMCI) guidelines provide the basis for treatment recommendations for children with pneumonia in most low-resource settings [1–3]. The IMCI guidelines were written for doctors, nurses, and other nonphysician clinicians working at first-level outpatient facilities in low-resource settings such as clinics, health centers, or outpatient departments of hospitals [1–3]. In contrast, the WHO integrated community case management guidelines were envisioned to target lay, informally trained healthcare providers, often called community health workers, who staff community-level village clinics or conduct household-level care [4]. The recommendations detailed in this Viewpoint are specific to the IMCI guidelines and the appropriate healthcare providers and health system levels for which these guidelines were intended.

IMCI implementation, dating back 2 decades, has contributed to global child pneumonia mortality reductions of >30% [5]. Despite laudable progress, pneumonia remains the leading cause of death in children aged 1–59 months worldwide [5]. IMCI recommends home oral amoxicillin treatment, rather than hospitalization and parenteral antibiotics, for human immunodeficiency virus (HIV)–uninfected children 2–59 months of age with cough and/or difficult breathing, chest indrawing, and no general danger signs (chest indrawing pneumonia) [1]. Several multicountry randomized controlled trials that reported equivalent outcomes between oral and parenteral antibiotics for children with chest indrawing pneumonia provided evidence supporting this recommendation [6–9]. Translating these trial results into widescale programmatic care poses significant challenges as there are several key issues that limit the generalizability of these trials to real-world settings. Unlike appropriately resourced and supervised clinical trials, effective guideline implementation in real-world settings hinges upon often inadequately trained, poorly supervised, and underresourced healthcare workers to correctly identify chest indrawing and exclude any accompanying clinical danger signs and underlying chronic illnesses that require hospitalization. We will discuss these issues here in this Viewpoint, revisiting evidence from the original studies that formed the basis of these guidelines to offer our alternative perspective. We will also review the physiology of chest indrawing and examine the risks, alongside proposed solutions, of implementing these guidelines in frontline outpatient facilities in low-resource settings.

Chest indrawing, as defined by the WHO IMCI guidelines, is the abnormal inward movement of subcostal tissue (ie, the tissue inferior to the costal cartilage of the lower anterior chest wall) during inspiration [2, 3], and in children, chest indrawing often occurs during respiratory diseases with poorly compliant, or “stiff,” lungs. During pneumonia, lung compliance, or the change in volume per unit change in pressure, decreases as airway and alveolar inflammation progresses [10]. To maintain adequate tidal volume respirations during diseases with low lung compliance, greater inspiratory force must be generated (ie, more negative intrapleural pressures). Generating more negative intrapleural pressures during inspiration can pull the subcostal tissue inward, producing what the WHO IMCI guidelines define as chest indrawing. The need to generate even more negative intrapleural pressures secondary to worsening lung compliance may also require the use of accessory muscles of respiration such as the intercostal, sternocleidomastoid, and scalene muscles located between the ribs and the lateral neck, respectively. Accessory respiratory muscle use can produce additional signs of severe respiratory distress such as head nodding (sternocleidomastoid and scalene muscle contraction), tracheal tugging (sternocleidomastoid and scalene muscle contraction), and intercostal recessions (external intercostal muscle contraction). See Table 1 for more detailed descriptions of the signs of severe respiratory distress included in this Viewpoint. Given the association between chest indrawing, low lung compliance, and pneumonia, and the fact that chest indrawing can be observed without additional equipment, the WHO utilizes chest indrawing as a diagnostic sign for pneumonia.

**Table 1.** Definitions for Signs of Severe Respiratory Distress

Sign	Description
Grunting	Repetitive “eh” sounds, usually short in duration, during early expiration against a partially closed glottis. Represents the child’s attempt to generate additional positive end expiratory pressure and maintain lung volume.
Nasal flaring	Consistent and repetitive outward movement of the ala nasi (lateral aspect of the nares) during inspiration. Represents the child’s attempt to reduce inspiratory resistance and ease overall breathing effort.
Head nodding	The head consistently moves upward and downward in synchrony with respiration. Occurs in young children who have limited head control, due to the bilateral retraction and relaxation of the sternocleidomastoid and scalene muscles of the lateral neck during respiration. This sign is most visible in the upright position and least visible if the child’s head is supported.
Tracheal tugging	The soft tissue over the trachea immediately superior to the sternum consistently pulls inward during inspiration. Can occur in younger or older children due to a combination of the bilateral retraction and relaxation of the sternocleidomastoid and scalene muscles of the lateral neck during respiration, and the more negative intrapleural pressures generated during inspiration, in an effort to maintain tidal volumes during low lung compliance states.
Intercostal retractions	The tissue between the ribs consistently pulls inward during inspiration. Occurs due to the retraction of the external intercostal muscles during inspiration, and also from the more negative intrapleural pressures generated during inspiration, in an effort to maintain tidal volumes during low lung compliance states.
Very fast breathing for age (severe tachypnea)	A child aged 2–11 months breathing at ≥70 breaths/min or a child aged 12–59 months breathing at ≥60 breaths/min. When attributable to lung disease, this occurs as a compensatory response to maintain minute ventilation when tidal volumes are compromised. This can also occur from other causes that increase the respiratory rate such as fever, anxiety, pain, dehydration, and sepsis.

Chest indrawing in children <2 years of age requires separate consideration, as chest indrawing in this age range is less specific for pneumonia, especially when it is observed alone without signs of severe respiratory distress (ie, grunting, nasal flaring, head nodding, tracheal tugging, intercostal retractions, severe tachypnea). Chest indrawing’s decreased specificity in younger children relates to chest wall skeletal maturation and respiratory system compliance (ie, the sum of lung and chest wall compliances) [11]. In younger children, the chest wall is nearly 3 times more compliant than the lungs due to immature bone ossification. This imbalance lowers the overall respiratory compliance, even in a healthy pulmonary system, due to the lung’s natural tendency to recoil [11]. During the first 2 years of life, as the bones ossify, chest wall compliance falls linearly until equilibrating with the lungs, which occurs in most children by the second year of life [11]. Therefore, even without pulmonary disease, younger children ventilate at a mechanically disadvantageous state, requiring comparatively more negative intrapleural pressures to maintain sufficient tidal volumes for gas exchange [11]. As a result, chest indrawing can be observed in children <2 years of age without lower respiratory disease, such as those with mildly increased upper airway resistance from nasopharyngeal inflammation, for example, or nonrespiratory diseases with high metabolic demands that increase minute ventilation, such as febrile illnesses.

While in younger children chest indrawing can be less specific and nonsevere, its occurrence in the presence of signs of severe respiratory distress or hypoxemia increases its specificity for pulmonary disease and substantially alters a child’s mortality risk profile. The WHO IMCI guidelines do not address signs of severe respiratory distress or sufficiently emphasize the importance of noninvasive peripheral oxygen saturation (SpO_2_) measurement by pulse oximetry [1–3]. Chest indrawing in combination with signs of severe respiratory distress is likely to represent the body’s attempt to compensate for an even greater reduction in lung compliance. Signs of severe respiratory distress indicate a higher likelihood of respiratory decompensation, as is suggested by the association of these signs with severe hypoxemia (SpO_2_ <90%), a key predictor of child pneumonia mortality [12]. While the WHO Pocketbook of Hospital Care for Children, which are guidelines written for doctors, senior nurses, and nonphysician clinicians practicing at first-referral level hospitals, account for this added risk by incorporating signs of severe respiratory distress into the criteria for severe pneumonia requiring hospitalization [13], the WHO IMCI guidelines do not [1–3]. The WHO IMCI guidelines infer that the mortality risk of all chest indrawing illnesses are equivalent, even if signs of severe respiratory distress are observed.

Reliably identifying respiratory signs, whether severe or not, can be difficult, as accurate identification requires additional healthcare worker training and supervision that is not routinely available in most low-resource settings. Respiratory signs can be subtle, infrequent, and variably present, even during single patient encounters, particularly if the child is agitated with an unstable respiratory pattern. Agitated breathing is common during busy, anxiety-provoking clinical environments and can distort respiratory patterns, and may lead healthcare providers to miss nonsevere or severe respiratory signs. In one study, pediatricians failed to successfully count the respirations in 16% of agitated children, compared to only 6%–8% of children who were either awake, feeding, or sleeping (*P* < .01) [14]. Investigators studying pediatric respiratory patterns at a Tanzanian outpatient clinic found an agitated state to be independently associated with a greater variation of respiratory rates over a 60-minute period of observation, concluding that respiratory examinations in busy, noisy clinics may be unreliable and can misclassify pneumonia cases [15]. Calming ill children so that their breathing patterns can be more accurately observed requires training, practice, and patience. Despite these drawbacks, IMCI’s exclusion of signs of severe respiratory distress as indications for referral and/or closer monitoring increases the likelihood of misclassification and less vigilant treatment of higher-risk children.

Children with pneumonia and chronic illnesses are at greater mortality risk but were largely excluded from previously mentioned trials on which the current guidelines are based, likely underestimating true treatment failure rates among children with chest indrawing pneumonia during routine programmatic conditions [6–9]. Twelve studies from low-resource settings included in a meta-analysis of child pneumonia mortality risk factors reported a pooled odds ratio (OR) for death of 4.76 (95% confidence interval [CI], 3.27–6.93) among children with chronic diseases and pneumonia [16]. Typically, children with chronic illnesses present to care similarly to those without chronic disease, and are therefore difficult to reliably identify without diagnostic support. Outpatient clinics and health centers in low-resource settings, the health system levels targeted by IMCI, characteristically have limited available diagnostics. This inability to reliably identify children with chronic illnesses at frontline outpatient facilities poses a threat to successful implementation of these IMCI recommendations. HIV is one example of a chronic illness that requires exclusion in children with chest indrawing pneumonia in HIV-endemic settings.

In HIV-endemic African countries, HIV infection and exposure remain disproportionally prevalent in children with pneumonia and are drivers of poor outcomes. A single-cause mortality model estimated that a median of 27% of all pneumonia deaths (interquartile range, 14%–47%) among children aged 1–59 months in 2010 could be attributable to HIV in the 22 HIV priority countries of the Joint United Nations Programme on HIV/AIDS (UNAIDS) Global Plan, 21 of which were in Africa [17]. Overall, child pneumonia deaths attributable to HIV constitute about 10% of all child pneumonia deaths globally [17]. Misclassifying HIV-infected or -exposed children as low risk is an important potential pitfall of current IMCI recommendations, as pediatric HIV testing programs continue to underperform. In 2012, for example, only about one-third of children born to HIV-infected mothers received an HIV test in the first 2 months of life [17]. Indeed, in one of the trials that provided evidence for the current WHO chest indrawing pneumonia recommendations, an interim analysis found a trend toward elevated mortality in South Africa and Zambia where HIV prevalence was high [7]. Citing concerns that some children with HIV were unrecognized and incorrectly enrolled, the study’s safety committee expanded exclusion criteria to better identify those with suspected HIV. The study ultimately reported more frequent treatment failure in a subset of enrolled children with HIV, regardless of age and treatment [7]. Currently, IMCI does not address the most common scenario of chest indrawing pneumonia management as it relates to pediatric HIV, that is, whether a child with an *unknown* HIV status in an HIV-endemic setting should or should not be referred or more closely monitored.

Translating trials into implementable guidelines is an incremental process, and feasible solutions exist to address these gaps (Table 2). First, in addition to harmonizing the WHO IMCI guidelines with the WHO Pocketbook of Hospital Care for Children to include signs of severe respiratory distress as warranting consideration of referral and/or closer monitoring, evidence exists to support also including “moderate” malnutrition as a possible referral indication and severe tachypnea (ie, very fast breathing for age) as another sign of severe respiratory distress. Multiple studies from low-resource settings have identified moderate malnutrition as associated with increased mortality risk among children with pneumonia. Eighteen studies included in a meta-analysis examining risk factors for child pneumonia mortality in low-resource settings reported a pooled OR for death of 2.46 among those with pneumonia and moderate malnutrition (95% CI, 1.89–3.19) [16]. A study from Malawi used mid-upper arm circumference measurements to nutritionally assess children 2–59 months old with WHO pneumonia and found that those with measurements between 115 and 135 mm, “moderate malnutrition,” had an increased OR of death of 1.73 in adjusted models (95% CI, 1.21–2.48) [18]. Of note, malnutrition can be a reasonable proxy for chronic illness given that most chronic diseases are associated with increased metabolic demands that worsen baseline nutritional states. The landmark chest indrawing clinical trials also demonstrated that severe tachypnea (≥70 breaths/minute for children aged 2–11 months and ≥60 breaths/minute for children aged ≥12 months) was associated with greater treatment failure risk, reporting pooled adjusted ORs for treatment failure between 2.0 and 6.9, depending upon the age cutoff used [6]. Routinely measuring mid-upper arm circumference for moderate malnutrition and using higher respiratory rate cutoffs for consideration of hospital referral and/or closer monitoring could be feasibly implemented in outpatient clinics in low-resource settings.

**Table 2.** Proposed Modifications to World Health Organization (WHO) Integrated Management of Child Illness Guidelines for Children Aged 2–59 Months With WHO Chest Indrawing Pneumonia at Outpatient Health Facilities

Consider referral and/or daily monitoring if referral is not possible with any of the following:
1. Pulse oximetry
a. SpO_2_ <93% (at altitudes <2000 m)
b. Facility lacks the capacity to perform pulse oximetry or the SpO_2_ measurement could not be obtained for other reasons
2. Signs of severe respiratory distress
a. Grunting
b. Nasal flaring
c. Head nodding
d. Tracheal tugging
e. Intercostal retractions
f. Very fast breathing for age (severe tachypnea)
o ≥70 breaths/min for children aged 2–11 mo
o ≥60 breaths/min for children aged 12–59 mo
3. Moderate malnutrition
a. Mid-upper arm circumference 115–135 mm
b. Weight-for-age or weight-for-height *z* score –2 to –3
c. Facility lacks the capacity to perform malnutrition assessment and nutritional status is unknown or not recently assessed
4. HIV testing recommendations for HIV-endemic settings: Facility lacks the capacity to perform HIV testing and the HIV status is unknown, or not recently assessed if the child is breastfeeding

Abbreviations: HIV, human immunodeficiency virus; SpO_2_, peripheral oxygen saturation.

Second, the WHO IMCI guidelines do not sufficiently emphasize routine SpO_2_ measurement with pulse oximeters, recommending pulse oximetry only if available [1]. Studies have consistently shown that an SpO_2_ <90% is predictive of mortality among children with pneumonia [18, 19], with 1 meta-analysis reporting a pooled OR of death of 5.47 (95% CI, 3.93–7.63) from 13 studies of children with clinical pneumonia and SpO_2_ <90% in low-resource settings [20]. One study from Malawi further heightens concern, reporting that outpatient treatment of chest indrawing pneumonia without pulse oximetry would miss more than two-thirds of children with SpO_2_ <90% at frontline outpatient health facilities [21]. The same meta-analysis also reported a pooled OR of death of 3.66 (95% CI, 1.42–9.47) from 3 studies of children with clinical pneumonia and SpO_2_ <93% [20]. A more recent study, also from Malawi, specifically focused on an SpO_2_ range of 90%–92%, “moderate hypoxemia,” among children with WHO pneumonia and found this to be independently predictive of mortality (OR, 1.54 [95% CI, 1.05–2.28]) [18]. These studies lend supportive data to an SpO_2_ threshold of <93% as a potential referral indication when not at high altitude. Last, as mentioned, reliably identifying respiratory signs, especially signs of severe respiratory distress, is a challenge in children. Given that signs of severe respiratory distress are associated with hypoxemia [12], SpO_2_ can serve as an aid to improve the detection of children with these signs, even if the healthcare provider misses them. SpO_2_ screening as early in the patient care pathway as is feasible, ideally during triage, can minimize misclassification of hypoxemic children and better optimize care. Research from Malawi demonstrated that outpatient healthcare provider cadres measured SpO_2_ values, compared to a pediatrician’s reference measurement, with the same reliability as those healthcare providers working at hospitals, and were able to successfully obtain an SpO_2_ in 93% of approximately 6500 children with WHO pneumonia [21]. Taken together, these data suggest that the performance of outpatient healthcare providers and the reliability of their pulse oximetry measurements should be no different than SpO_2_ measurements obtained at higher levels of the health system. IMCI should be revised to recommend routine SpO_2_ screening, preferably during initial triage, with consideration of referral and/or closer monitoring of any child not at high altitude with SpO_2_ <93%.

We acknowledge that retraining healthcare providers targeted by the WHO IMCI guidelines to reliably recognize signs of severe respiratory distress and develop skills to successfully calm agitated children are legitimate challenges, from both a training and funding perspective, and that the implementation of any guideline revision, including pulse oximetry and MUAC measurement, requires the reeducation of existing healthcare providers and updating of healthcare institution curricula. This is no small feat. However, in the internet era, where online training curricula are commonly used for competency-based adult learning and ongoing medical education, we believe that there is sufficient educational and technical expertise to modernize the WHO’s process of guideline updates in order to execute a streamlined, efficient approach to guideline reeducation. This retraining barrier is both surmountable and worthy of investment. One recent example of this is from the Pneumonia Etiology Research for Child Health (PERCH) study where investigators developed an innovative, online, competency-based educational tool that used interactive videos and quizzes to successfully educate, standardize, and remotely monitor the performance of study personnel required to reliably recognize signs of severe respiratory distress, assess SpO_2_ with pulse oximetry, and measure MUACs in 7 study sites in Africa and Asia [22, 23]. These study personnel had the same educational background as those targeted by IMCI—that is, doctors, nurses, and nonphysician clinicians. This tool is now publically available and offers an important example of how to modernize the implementation and supervision of guideline revisions in the future.

In sum, we recommend the expedited revision of the WHO IMCI guidelines to better stratify mortality risk among children 2–59 months of age with chest indrawing pneumonia at frontline outpatient facilities. The presence of signs of severe respiratory distress (ie, grunting, nasal flaring, head nodding, tracheal tugging, intercostal retractions, severe tachypnea), SpO_2_ <93%, or moderate malnutrition should prompt consideration of referral and/or daily monitoring, as all are associated with increased risk of adverse outcomes. When a clinic lacks pulse oximetry or nutritional assessment tools, or lacks the capacity to conduct HIV testing in an HIV-endemic setting, we also recommend considering referral to complete the assessment. When referral is not possible in any scenario, including when referral hospitals or clinics are unable to accommodate additional transfers due to a lack of physical space or human resources, we recommend daily follow-up to monitor children for disease progression and treatment modification. To address case severity misclassification, we recommend SpO_2_ screening at the earliest feasible point in the care pathway at outpatient facilities, prioritizing children with respiratory presentations, including chest indrawing, or clinical danger signs.

## Notes

***Author contributions.*** E. D. M. wrote the first draft of the comment. E. D. M. and A. S. G. provided critical review and editing of comment. E. D. M. and A. S. G. reviewed subsequent drafts and agreed on the final version of this comment.

***Financial support.*** E. D. M. received support from the National Institutes of Health through the Fogarty International Center (grant number K01TW009988).

***Potential conflicts of interest.*** All authors: No reported conflicts of interest. Both authors have submitted the ICMJE Form for Disclosure of Potential Conflicts of Interest. Conflicts that the editors consider relevant to the content of the manuscript have been disclosed.
